# Timing of tourniquet release in total knee arthroplasty: A meta-analysis

**DOI:** 10.1097/MD.0000000000006786

**Published:** 2017-04-28

**Authors:** Pei Zhang, Yuan Liang, Jinshan He, Yongchao Fang, Pengtao Chen, Jingcheng Wang

**Affiliations:** a Department of Orthopedics, Clinical Medical College of Yangzhou University, Subei People's Hospital, Yangzhou; b Dalian Medical University, Dalian, Liaoning, China.

**Keywords:** arthroplasty, knee, meta-analysis, tourniquet

## Abstract

**Background:**

For total knee arthroplasty (TKA), the tourniquet is routinely employed for better visualization, less blood loss, and easier cementation. However, the time to release tourniquet remains controversial. Therefore, we performed current meta-analysis to assess whether releasing tourniquet before wound closure is more effective in reducing blood loss than releasing tourniquet after wound closure in TKA without an increased risk of complications.

**Methods:**

To conduct this meta-analysis, we searched Medline, Embase, Web of science, and the Cochrane library up to November 2016, for randomized controlled trials comparing tourniquet releasing before and after wound closure in TKA. A meta-analysis was performed following the guidelines of the Cochrane Reviewer's Handbook and the PRISMA statement. Methodological quality of the trials was assessed using the Cochrane risk assessment scale. The data of the included studies were analyzed using Stata 12.0.

**Results:**

Sixteen trials involving 1010 patients were identified in current meta-analysis. Our meta-analysis demonstrated that there were no significant differences in the 2 groups in terms of calculated blood loss (weighted mean difference [WMD] = 160.65, 95% confidence interval [CI]: −0.2 to 321.49, *P* = .05), postoperative blood loss (WMD = −45.41, 95% CI: −120.11 to 29.29, *P* = .233),postoperative hemoglobin decline (WMD = 0.16, 95% CI: −2.5 to 2.82, *P* = .905), transfusion volume (WMD = 79.19, 95% CI: −5.05 to 163.44, *P* = .065),transfusion rates (relative risk [RR] = 1.19, 95% CI: 0.95–1.50, *P* = .134), major complications (RR = 0.51, 95% CI: 0.15–1.73, *P* = .278), and deep vein thrombosis (RR = 0.44, 95% CI: 0.14–1.37, *P* = .157).Compared with the group of releasing tourniquet after wound closure, the group of releasing tourniquet before wound closure had a higher volume of total blood loss (WMD = 130.96, 95% CI: 58.83–203.09, *P* = .000) and a longer operation time (WMD = 6.56, 95% CI: 3.12–10.01, *P* = .000). However, releasing tourniquet before wound closure could reduce minor complications (RR = 0.53, 95% CI: 0.34–0.82, *P* = .004).

**Conclusions:**

On the basis of current meta-analysis, the method of releasing tourniquet before wound closure could increase total blood loss and operation time; nevertheless, the risk of complications decreased. Thus, if patients are in severe anemia condition, the tourniquet perhaps should be released after wound closure to decrease blood loss. In contrary, releasing tourniquet before wound closure to decrease the risk of complications would be a better choice.

## 1 Introduction

Although a variety of complications are described for the use of pneumatic tourniquet, such as neuromuscular injuries, increased postoperative pain, delayed wound healing, increased thrombotic events,^[1–5]^ the tourniquet is routinely employed for better visualization, less blood loss, and easier cementation in total knee arthroplasty (TKA).^[6]^ To reduce the tourniquet time and the incidence rate of complications, some surgeons suggested to release tourniquet before wound closure, which has been reported to have less blood loss, lighter postoperative pain, lower incidence rate of complication, better evaluation of patellar tracking, and faster functional recovery.^[7–10]^ Although many studies involving randomized controlled trials (RCTs), retrospective studies, and systematic review^[7,8,10–26]^ have investigated whether releasing tourniquet before wound closure is more effective in reducing blood loss than releasing tourniquet after wound closure in TKA without an increased risk of complications, it is still highly debatable. Therefore, we conducted current meta-analysis which just included RCTs to prove it.

## 2 Materials and methods

We conducted this study according to the methods of the Cochrane Handbook5.1 and we reported our findings according to PRISMA Statement.

### 2.1 Literature search

Literature searches of Medline, EMBASE, Web of Science, and the Cochrane Library were performed up to November 2016. References of each study were checked for potentially relevant studies. There were no language restrictions. When it was necessary, the authors of the included articles were contacted for original data. The pooling of data was carried out using stata12.0. The key words were used including “randomized controlled trials,” “tourniquet,” “total knee replacement/arthroplasty.” We combined them with Boolean operators.

### 2.2 Inclusion and exclusion criteria

We identified literature that met the following inclusion criteria: RCTs that comparing tourniquet releasing before and after wound closure during TKA surgery; the results of studies included at least one of the outcome measures (blood loss: total blood loss, calculated blood loss, postoperative blood loss, postoperative hemoglobin decline, transfusion volume, transfusion rates, major complication, minor complications, DVT, and operation time).

Exclusion criteria were different tourniquet application strategy was used; not a RCT; duplicate publication.

### 2.3 Data extraction

Two authors (YL and PC) independently extracted the data according to our early designed data extraction form, including publication information (authors, publication year), targeted population, group size, average age, anticoagulant, drainage, tourniquet pressure, and tourniquet time; clinical outcomes (blood loss: total blood loss, calculated blood loss, postoperative blood loss; postoperative hemoglobin decline; transfusion volume; the number of transfused); complications (minor complications, major complications, the number of DVT); and operation time. Concerning complications, we described the complication as a major one if a second operation under anesthesia (drainage, debridement, and even a revision) was needed, such as vessels injury, wound dehiscence, active hemorrhage, severe hematoma, deep infection, and so on. Minor complications were superficial infection, marginal necrosis, wound oozing, leg swelling, minor wound dehiscence, DVT, and so on, which could be healed by conservative treatment. Whenever discrepancies existed, another investigator (JH) was consulted to solve them.

### 2.4 Quality assessment

Two investigators (YL and PC) assessed the quality of the RCTs according to the method in the Cochrane Reviewer's Handbook 5.1.0 independently. The risk of bias of the included studies was assessed according to the Cochrane risk assessment scale, including the following contents: details of the methods of random sequence generation, allocation concealment, blinding, incomplete outcome data, selective outcome reporting, and other sources of bias. If disagreements existed, they were resolved by discussing with another investigator (YF).

### 2.5 Data analysis and statistical methods

The meta-analysis was carried out using Stata 12.0. For continuous outcomes, we calculated the weighted mean difference (WMD) with 95% confidence interval (CI), and the relative risk (RR) with 95% CI was calculated for dichotomous data. Random-effects models were used to reduce heterogeneity. If necessary, sensitivity analysis was conducted to identify the origins of the significant heterogeneity. Publication bias was assessed concerning total blood loss in current meta-analysis because only the outcome total blood loss was reported in at least 10 studies. Besides that, we conducted subgroup analysis based on fixation type to explore some possible differences.

## 3 Results

### 3.1 Literature search

**Figure 1:**
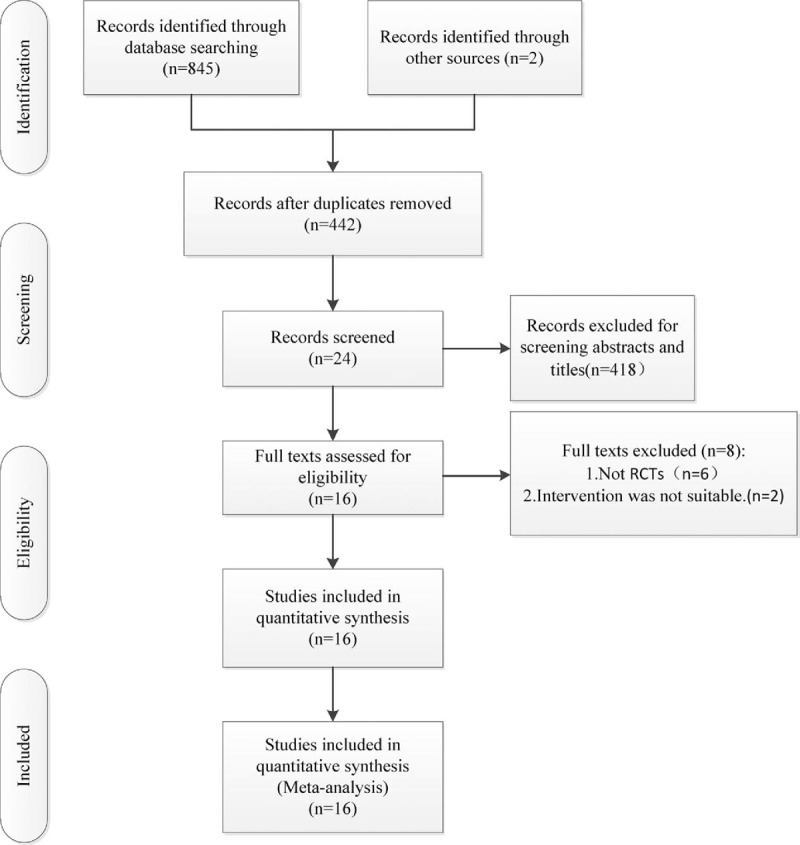
The flow chart of literature screening.

We just included randomized controlled trials comparing tourniquet releasing before or after wound closure in TKA. Retrospective and prospective quasiexperimental studies were excluded. A total of 845 potentially relevant studies were initially retrieved. By scanning the titles and abstracts of each study, 418 studies were excluded from analysis. After full texts were assessed for eligibility, 6 studies^[11,27–31]^ were excluded because they were not RCTs, 2 studies were excluded because of different intervention.^[32,33]^ After reviewing bibliographies of each study, 2 studies^[12,25]^ were found. Finally, 16 RCTs^[8,10,12–25]^ were included in our meta-analysis. The flow chart of study selection is shown in Figure 1 and the characteristics of each study are shown in Table 1.^[8,10,12–25]^

**Table 1:**
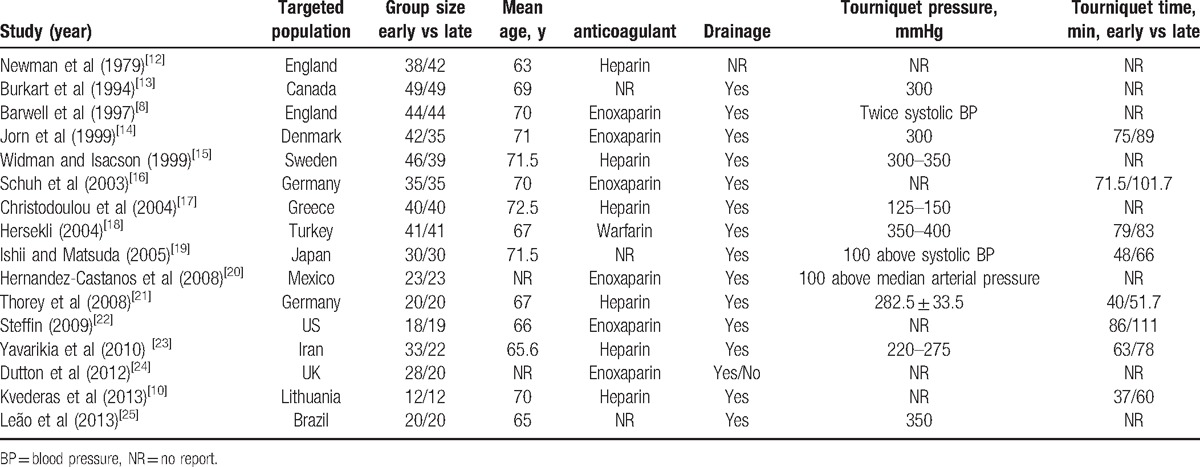
The characteristics of included studies.

### 3.2 Risk of bias assessment

**Figure 2:**
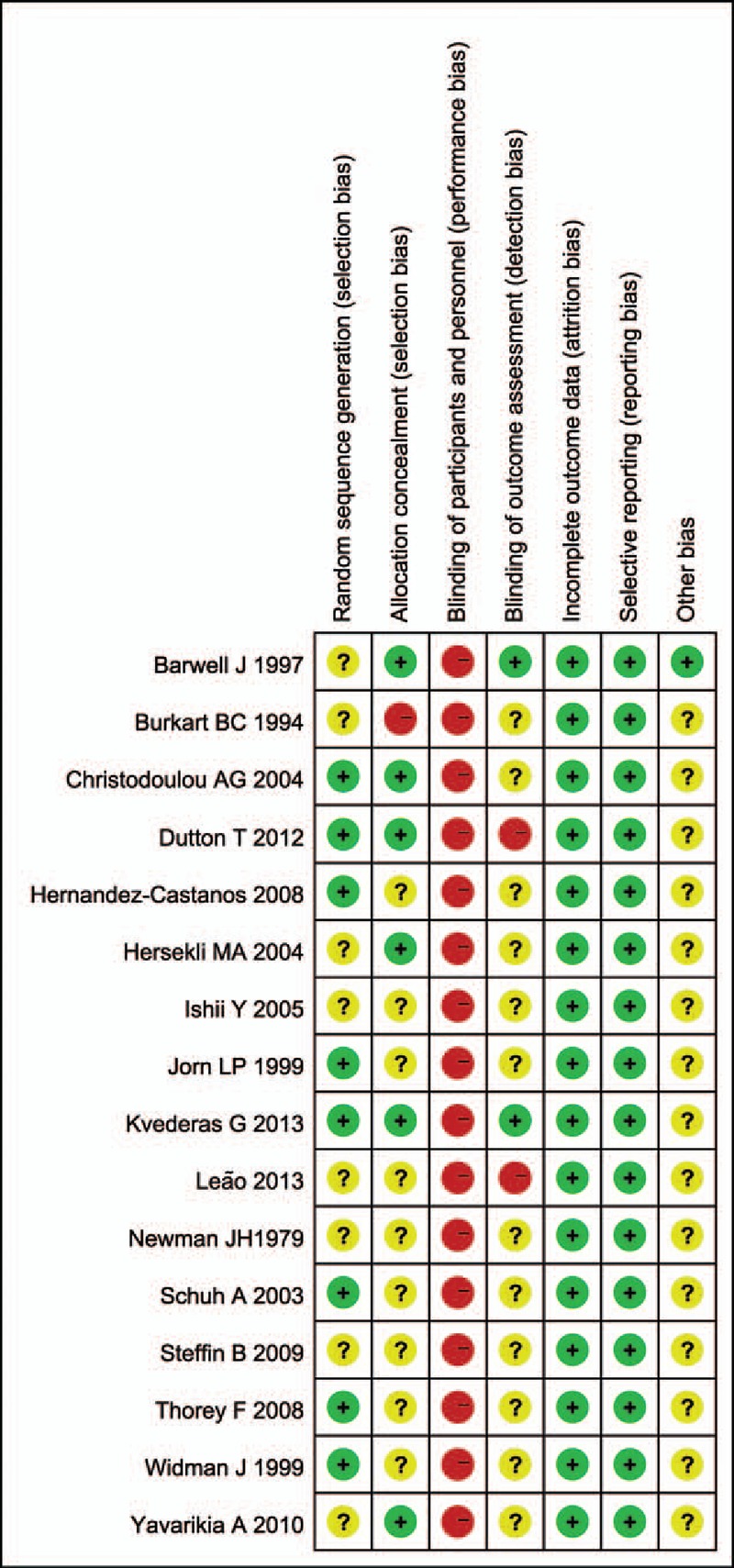
Risk of bias summary.

In 8^[10,14–17,20,21,24]^ of the RCTs, the randomization algorithm was generated from a computer or blinded statistician. In 6 of the RCTs,^[8,10,17,18,23,24]^ the allocation concealment was achieved by sealed envelope system. None of them provided the information of double blinding. Only 2^[8,10]^ of the RCTs involved blinding of outcome assessment. However, all of the RCTs were reported with complete outcome data and no selective outcome reporting. The absolute result is shown in Figure 2.

### 3.3 Outcomes measure

#### 3.3.1 Blood loss (milliliters)

**Figure 3:**
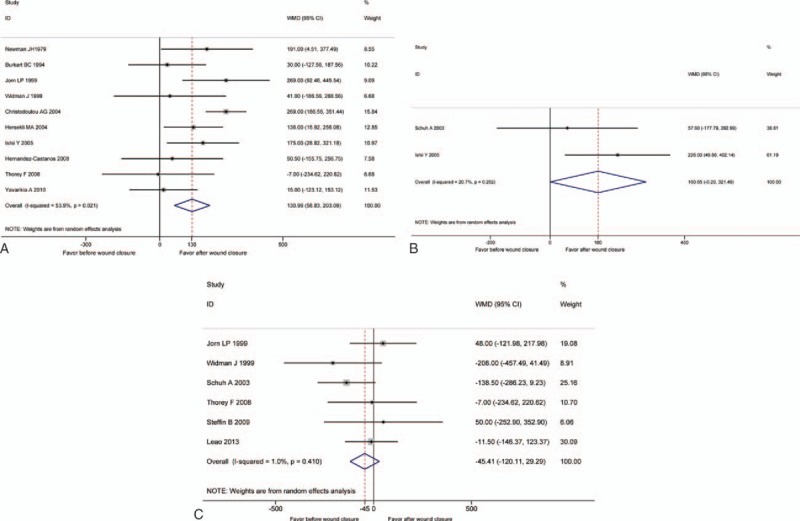
(A) The comparison of total blood loss. (B) The comparison of calculated blood loss. (C) The comparison of postoperative blood loss.

Ten studies^[12–15,17–21,23]^ compared total blood loss. The pooled result manifested that the group of releasing tourniquet before wound closure had a higher volume of total blood loss (WMD = 130.96, 95% CI: 58.83–203.09, *P* = .00, *I*^2^ = 53.9%) **(**Fig. 3A) Subgroup analysis demonstrated similar results between the 2 groups concerning cemented fixation (WMD = 91.49; 95% CI: 19.57–163.41; *P* = .013; *I*^2^ = 0%).

Two studies^[16,19]^ provided data on calculated blood loss. No significant difference was found in calculated blood loss between the 2 groups (WMD = 160.65, 95% CI: −0.2 to 321.49 *P* = .05, *I*^2^ = 20.7%) (Fig. 3B). It was a trend releasing tourniquet before wound closure had a higher volume of calculated blood loss. The lack of data may contribute to it.

Six studies^[14–16,21,22,25]^ compared postoperative blood loss. The pooled result demonstrated no significant difference between the 2 groups (WMD = −45.41, 95% CI: −120.11 to 29.29, *P* = .233, *I*^2^ = 1%) (Fig. 3C).

#### 3.3.2 Hemoglobin drop (gram per liters)

**Figure 4:**
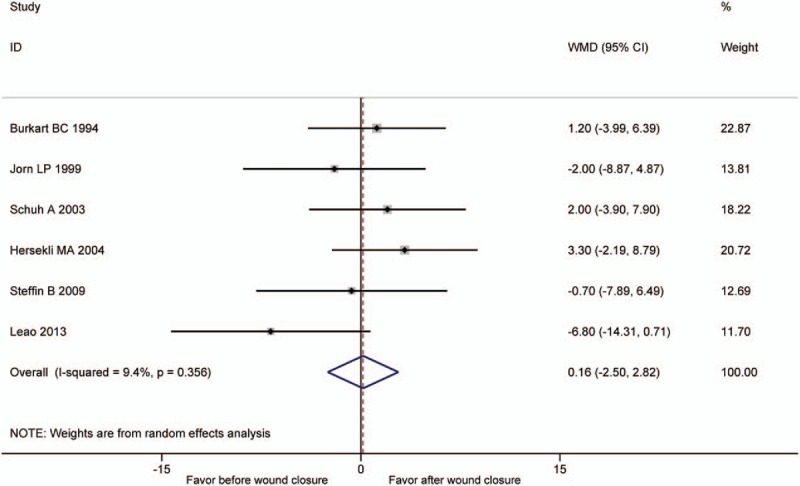
The comparison of postoperative hemoglobin decline.

Six studies^[13,14,16,18,22,25]^ included the comparison of hemoglobin drop. Pooling data demonstrated no significant difference between the 2 groups (WMD = 0.16, 95% CI: −2.5 to 2.82, *P* = .905, *I*^2^ = 9.4%) (Fig. 4).

#### 3.3.3 Volume of transfusion (milliliters)

**Figure 5:**
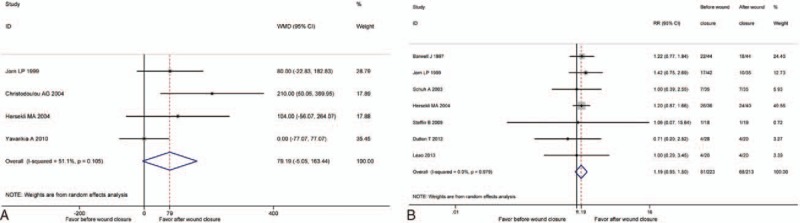
(A) The comparison of transfusion volume. (B) The comparison of transfusion rates.

Data on the volume of transfusion per patient were included in 4 studies.^[14,17,18,23]^ No significant difference was detected in the 2 groups (WMD = 79.19, 95% CI: −5.05 to 163.44, *P* = .065, *I*^2^ = 51.1%) (Fig. 5A).

### 3.4 Transfusions rate

Seven studies^[8,14,16,18,22,24,25]^ included the comparison of hemoglobin drop. Pooling data indicated no significant difference between the 2 groups (RR = 1.19, 95% CI: 0.95–1.50, *P* = .134; *I*^2^ = 0%) (Fig. 5B).

### 3.5 Major complications

**Figure 6:**
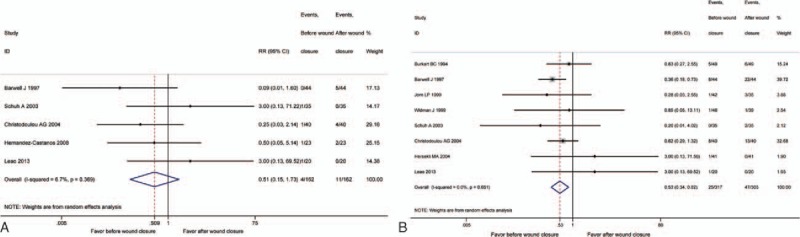
(A) The comparison of major complications. (B) The comparison of minor complications.

Data on the major complications were included in 5 studies.^[8,16,17,20,25]^ No significant difference was detected in the 2 groups (RR = 0.51, 95% CI: 0.15–1.73, *P* = .278, *I*^2^ = 6.7%) (Fig. 6A).

### 3.6 Minor complications

Eight studies^[8,13–18,25]^ compared postoperative blood loss. The pooled result demonstrated releasing tourniquet before wound closure could reduce minor complications (RR = 0.53, 95% CI: 0.34–0.82, *P* = .004, *I*^2^ = 0%) (Fig. 6B).

### 3.7 Thrombotic events (deep venous thrombosis)

**Figure 7:**
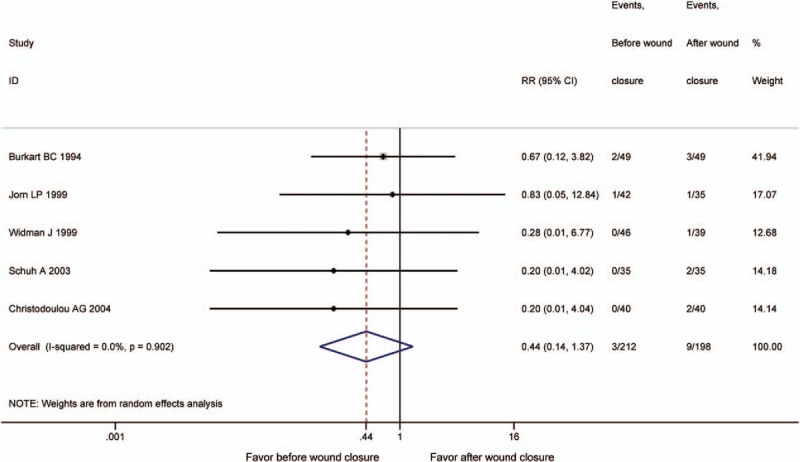
The comparison of deep vein thrombosis (DVT).

Deep venous thrombosis (DVT) events were reported in 5 studies.^[13–17]^ No significant difference was detected in the 2 groups (RR = 0.44, 95% CI: 0.14–1.37, *P* = .157, *I*^2^ = 0%) (Fig. 7).

### 3.8 Operative time (minutes)

**Figure 8:**
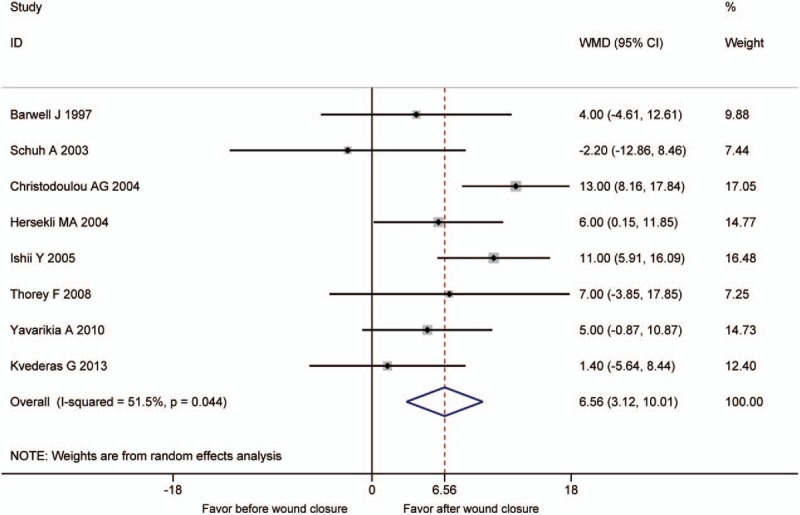
The comparison of operative time.

Data on the operative time were reported in 8 studies.^[8,10,16–19,21,23]^ The pooled result demonstrated the group of releasing tourniquet before wound closure had a longer operation time (WMD = 6.56, 95% CI: 3.12–10.01, *P* = .000, *I*^2^ = 51.5%) (Fig. 8).

### 3.9 Publication bias

Publication bias is generally performed only when at least 10 studies are included in the meta-analysis. Thus, total blood loss as an outcome in most of the studies was selected as an example. Egger test was used to test publication bias. The test showed publication bias existed in the meta-analysis of total measured blood loss (*P* = .048), and the picture showed the same result.

## 4 Discussion

Current meta-analysis demonstrated that there were no significant differences in calculated blood loss, postoperative blood loss, hemoglobin drop, volume of transfusion, transfusion rates, major complications, and DVT between tourniquet release before wound closure for hemostasis and tourniquet release after wound closure in TKA. The group releasing tourniquet before wound closure had a higher volume of total blood loss and a longer operation time. However, releasing tourniquet before wound closure could reduce minor complications. Our meta-analysis just included RCTs that made our conclusion much more credible. Besides that, there were much more outcomes in our meta-analysis.

Limiting blood loss is an important issue in TKA. Although it is controversial, a tourniquet is still widely used as a routine practice. Now, 2 major tourniquet application strategies are used: release tourniquet before or after wound closure. Some surgeons suggested to release tourniquet before wound closure, which has been reported to have less blood loss, lighter postoperative pain, lower incidence rate of complication, better evaluation of patellar tracking, and faster functional recovery.^[7–10]^ However, some surgeons recognized releasing tourniquet after wound closure as a better method.^[12–16,19,21,23,25]^

For blood loss, current meta-analysis found that releasing tourniquet before wound closure could increase total blood loss, but there was no increase in postoperative blood loss and calculated blood loss. Therefore, the intraoperative blood loss may contribute to the majority of increased blood loss. Erskine et al^[34]^ also concluded that the blood loss was associated with perioperative blood loss. Some previous studies favored our result that releasing tourniquet before wound closure had a higher volume of total blood loss.^[10,12,14,17–19]^

Releasing tourniquet before wound closure theoretically could ensure a better view of hemostasis, and patients would have better blood conservation. Nevertheless, there is a pronounced rise in fibrinolytic activity after the release of an arterial tourniquet,^[12,35,36]^ contributing to the higher perioperative blood loss; however, it could be controlled after wound pressure dressing application.^[12]^ In addition, it was impossible to find all bleeding points. Longer surgical time also contributed to the results of more perioperative blood loss in the group tourniquet releasing before wound closure. Perioperative blood loss in TKA patients has been reported in association with drain clamping technique,^[37]^ application of compression dressing,^[38]^ intravenous antifibrinolytic therapy,^[39]^ tourniquet application time,^[33]^ and early rehabilitation programs.^[10]^ Besides that, an early use of continuous passive motion machines could increase blood loss.^[32]^ In addition, a good knowledge of vascular anatomy and fine surgical technique are crucial to limit perioperative loss.

The model of prosthesis fixation could influence blood loss during knee replacement. Some studies have reported that cementless procedures are generally related to a higher blood loss TKA.^[13,14,17,40,41]^ Much more blood loss from the cut cancellous bone could explain it. Therefore, we made a subgroup analysis on total blood loss; it demonstrated similar results between the 2 groups concerning cemented fixation.

Regarding hemoglobin drop, the hemoglobin should decrease proportionally with the blood loss theoretically. However, our meta-analysis showed no significant difference in postoperative hemoglobin decline. The result was in accordance with all included studies comparing hemoglobin drop.^[13,14,16,18,22,25]^ In our point of view, the small sample size, the variability in the blood transfusion rates, and the different criteria for transfusion among these studies may contribute to it.

Despite the differences in total blood loss, there were also no statistical differences in the amount of transfusions per patient and the transfusion rate between the 2 groups. However, all the included studies^[8,14,16,18,22,24,25]^ concerning transfusions rate showed the similar result, and most of included studies^[14,18,23]^ that had a comparison of the amount of transfusions per patient favored our result.

Some previous studies demonstrated that releasing tourniquet after wound closure had an increased risk of postoperative complications such as wound complications, deep infection, DVT, and so on.^[8,26,42]^ In our meta-analysis, there was no significant difference in major complications between the 2 groups. However, releasing tourniquet before wound closure could reduce minor complications. The tourniquet pressure and the time of tourniquet application have a great influence on the incidence rate of complications.^[43]^ Olivecrona et al^[44]^ reported that the incidence rate of complications increased if tourniquet pressure was >293 mmHg and they found patients with a cuff pressure of ≤225 mmHg had no postoperative infections and a lower rate of wound complications.^[43]^ Jorgensen et al^[45]^ considered that the risk of complications could be significantly reduced if the duration of tourniquet application was within 150 minutes. The relatively safe tourniquet pressure and tourniquet duration in most included studies may result in the lack of significant differences regarding major complication in our meta-analysis. However, it had a trend that releasing tourniquet before wound closure could decrease the incidence rate of major complication.

DVT is an important issue in TKA; thus, we set it as an indicator in our meta-analysis from complications specially. No significant difference was found between the 2 groups. Now, the incidence rate of DVT has decreased a lot using support stockings, early mobilization, and the administration of anticoagulant, which could explain it.

The studies involving the comparison of postoperative knee function are lacking. Three studies^[8,10,15]^ in the current meta-analysis mentioned it. Barwell et al^[8]^ randomized 88 knee replacements to 2 groups demonstrating that releasing tourniquet before wound closure could perform straight-leg raising significantly earlier than releasing tourniquet after wound closure, but no statistically significant was found in the mean range of flexion. Widman and Isacson^[15]^ reported that they found no statistical difference in range of movement at the first postoperative follow-up 2 to 3 months after surgery. Kvederas et al^[10]^ concluded that the inflation of a tourniquet before the skin incision and deflation after cementation in TKA could acquire faster functional recovery. Chang et al^[27]^ also observed better earlier functional recovery as well as subjective performance at early postoperative follow-ups (6 weeks) through a prospective cohort study. However, strong evidence is needed to draw a conclusion that releasing tourniquet before wound closure is more effective on postoperative knee functional outcomes in TKA.

The research of Barwell et al^[8]^ was the only study concerning postoperative pain; the pain scores for patients in releasing tourniquet before wound closure group were significantly lower than those of releasing tourniquet after wound closure group. Nevertheless, the conclusion needs further confirmations.

Although major vascular damage in TKA is very rare,^[32,46]^ the method releasing the tourniquet before wound closure could be recognized as a practical way to determine the major vascular damage in TKA. As most of the reported vascular complications during TKA were because of atherosclerotic vascular disease, intraoperative tourniquet release may not be indicated. We consider that touching the dorsal artery of the foot should be a routine to determine a major artery injury in TKA. Releasing the tourniquet before wound closure could decrease the incidence rate of complication to a certain extent, which is the greatest strength, and to tourniquet release after wound closure, the greatest advantage may be a significantly lower in total blood loss.

The limitations of current meta-analysis are following: weaknesses of some included RCTs (poor descriptions in methods of randomization, concealment of allocation, blinding of outcome assessment); some data in included studies without SD were not pooled to analyze; the patients in the studies differed widely, and thus we used random-effects model to reduce heterogeneity; the different operative and postoperative techniques include the tourniquet pressure, the timing of drain clamping, thrombotic prophylaxis, the type of postoperative compressive dressing, and the type of rehabilitation program, which could influence the clinical outcomes (However, there are not enough data to conduct subgroup analysis.); publication bias existed.

## 5 Conclusions

On the basis of current meta-analysis, the method of releasing tourniquet before wound closure could increase total blood loss and operation time; nevertheless, the risk of complications decreased. Thus, if patients are in severe anemia condition, the tourniquet perhaps should be released after wound closure to decrease blood loss. Otherwise, releasing tourniquet before wound closure to decrease the risk of complications would be a better choice.

## References

[1] Sapega AA Heppenstall RB Chance B Optimizing tourniquet application and release times in extremity surgery. A biochemical and ultrastructural study. J Bone Joint Surg 1985;67:303–14. 3968122

[2] Abdel-Salam A Eyres KS Effects of tourniquet during total knee arthroplasty. A prospective randomised study. J Bone Joint Surg Br 1995;77:250–3. 7706340

[3] Klenerman L Is a tourniquet really necessary for knee replacement? J Bone Joint Surg Br 1995;77:174–5. 7706327

[4] Worland RL Arredondo J Angles F Thigh pain following tourniquet application in simultaneous bilateral total knee replacement arthroplasty. J Arthroplasty 1997;12:848–52. 9458249 10.1016/s0883-5403(97)90153-4

[5] Horlocker TT Hebl JR Gali B Anesthetic, patient, and surgical risk factors for neurologic complications after prolonged total tourniquet time during total knee arthroplasty. Anesth Analg 2006;102:950–5. 16492857 10.1213/01.ane.0000194875.05587.7e

[6] Jiang FZ Zhong HM Hong YC Use of a tourniquet in total knee arthroplasty: a systematic review and meta-analysis of randomized controlled trials. J Orthop Sci 2015;20:110–23. 25373840 10.1007/s00776-014-0664-6

[7] Page MH Shepherd BD Harrison JM Reduction of blood loss in knee arthroplasty. Aust N Z J Surg 1984;54:141–4. 6588953 10.1111/j.1445-2197.1984.tb06705.x

[8] Barwell NJ Anderson G Hassan A The effects of early tourniquet release during total knee arthroplasty: a prospective randomised double-blind study. J Bone Joint Surg Br 1997;79B:265–8. 10.1302/0301-620x.79b2.7191 9119854

[9] Marson BM Tokish JT The effect of a tourniquet on intraoperative patellofemoral tracking during total knee arthroplasty. J Arthroplasty 1999;14:197–9. 10065726 10.1016/s0883-5403(99)90125-0

[10] Kvederas G Porvaneckas N Andrijauskas A A randomized double-blind clinical trial of tourniquet application strategies for total knee arthroplasty. Knee Surg Sports Traumatol Arthrosc 2013;21:2790–9. 23052115 10.1007/s00167-012-2221-1

[11] Huang Z Pei F Ma J Comparison of three different tourniquet application strategies for minimally invasive total knee arthroplasty: a prospective non-randomized clinical trial. Arch Orthop Trauma Surg 2014;134:561–70. 24515866 10.1007/s00402-014-1948-1

[12] Newman JH Jackson JP Waugh W Timing of tourniquet removal after knee replacement. J R Soc Med 1979;72:492–4. 552547 10.1177/014107687907200706 PMC1436934

[13] Burkart BC Bourne RB Rorabeck CH The efficacy of tourniquet release in blood conservation after total knee arthroplasty. Clin Orthop Relat Res 1994;147–52. 8119009

[14] Jorn LP Lindstrand A Toksvig-Larsen S Tourniquet release for hemostasis increases bleeding: a randomized study of 77 knee replacements. Acta Orthop Scand 1999;70:265–7. 10429602 10.3109/17453679908997804

[15] Widman J Isacson J Surgical hemostasis after tourniquet release does not reduce blood loss in knee replacement. A prospective randomized study of 81 patients. Acta Orthop Scand 1999;70:268–70. 10429603 10.3109/17453679908997805

[16] Schuh A Hausel M Salminen S Effect of tourniquet use on blood loss in total knee arthroplasty. Zentralbl Chir 2003;128:866–70. 14628238 10.1055/s-2003-44339

[17] Christodoulou A Ploumis A Terzidis I The role of timing of tourniquet release and cementing on perioperative blood loss in total knee replacement. Knee 2004;11:313–7. 15261219 10.1016/j.knee.2003.09.005

[18] Hersekli MA Akpinar S Ozkoc G The timing of tourniquet release and its influence on blood loss after total knee arthroplasty. Int Orthop 2004;28:138–41. 15024499 10.1007/s00264-004-0550-5 PMC3474495

[19] Ishii Y Matsuda Y Effect of the timing of tourniquet release on perioperative blood loss associated with cementless total knee arthroplasty: a prospective randomized study. J Arthroplasty 2005;20:977–83. 16376251 10.1016/j.arth.2005.01.012

[20] Hernandez-Castanos DM Ponce VV Gil F Release of ischaemia prior to wound closure in total knee arthroplasty: a better method? Int Orthop 2008;32:635–8. 17503044 10.1007/s00264-007-0376-z PMC2551729

[21] Thorey F Stukenborg-Colsman C Windhagen H The effect of tourniquet release timing on perioperative blood loss in simultaneous bilateral cemented total knee arthroplasty: a prospective randomized study. Technol Health Care 2008;16:85–92. 18487854

[22] Steffin B Green-Riviere E Giori NJ Timing of tourniquet release in total knee arthroplasty when using a postoperative blood salvage drain. J Arthroplasty 2009;24:539–42. 18534405 10.1016/j.arth.2008.01.302

[23] Yavarikia A Amjad G Davoudpour K The influence of tourniquet use and timing of its release on blood loss in total knee arthroplasty. Pak J Biol Sci 2010;13:249–52. 20464949 10.3923/pjbs.2010.249.252

[24] Dutton T De-Souza R Parsons N The timing of tourniquet release and ’retransfusion’ drains in total knee arthroplasty: a stratified randomised pilot investigation. Knee 2012;19:190–2. 21440444 10.1016/j.knee.2011.02.013

[25] Leão MGDS Souza HAPD Ferreira YMC Evaluation of blood loss after early or late release of ischemia in patients undergoing total knee replacement. Revbrasortop 2013;48:152–8. 10.1016/j.rboe.2012.03.003 PMC6565905 31211121

[26] Zan PF Yang Y Fu D Releasing of tourniquet before wound closure or not in total knee arthroplasty: a meta-analysis of randomized controlled trials. J Arthroplasty 2015;30:31–7. 25175846 10.1016/j.arth.2014.07.034

[27] Chang CW Lan SM Tai TW An effective method to reduce ischemia time during total knee arthroplasty. J Formos Med Assoc 2012;111:19–23. 22333008 10.1016/j.jfma.2012.01.006

[28] Urbano Manero EM Miguelena Bobadilla JM Timing of tourniquet release in total knee arthroplasty doesn’t affect on transfusion needs. Rev Esp Anestesiol Reanim 2012;59:556–61. 23084319 10.1016/j.redar.2012.07.020

[29] Abbas K Raza H Umer M Effect of early release of tourniquet in total knee arthroplasty. J Coll Physicians Surg Pak 2013;23:562–5. 23930872

[30] Yildiz C Koca K Kocak N Late tourniquet release and drain clamping reduces postoperative blood loss in total knee arthroplasty. HSS J 2014;10:2–5. 24482614 10.1007/s11420-013-9363-7 PMC3903953

[31] Hafeez S Amin MS Ameen J Early release of tourniquet in total knee arthroplasty: is it worthwhile? J Pak Med Assoc 2015;65:S77–81. 26878542

[32] Lotke PA Faralli VJ Orenstein EM Blood loss after total knee replacement. Effects of tourniquet release and continuous passive motion. J Bone Joint Surg Am 1991;73:1037–40. 1874765

[33] Wang K Ni S Li Z The effects of tourniquet use in total knee arthroplasty: a randomized, controlled trial. Knee Surg Sports Traumatol Arthrosc 2016. 10.1007/s00167-015-3964-2 26745962

[34] Erskine JG Fraser C Simpson R Blood loss with knee joint replacement. J R Coll Surg Edinb 1981;26:295–7. 7288695

[35] Klenerman L Chakrabarti R Mackie I Changes in haemostatic system after application of a tourniquet. Lancet 1977;1:970–2. 67466 10.1016/s0140-6736(77)92276-0

[36] Harvey EJ Leclerc J Brooks CE Effect of tourniquet use on blood loss and incidence of deep vein thrombosis in total knee arthroplasty. J Arthroplasty 1997;12:291–6. 9113543 10.1016/s0883-5403(97)90025-5

[37] Tai TW Yang CY Jou IM Temporary drainage clamping after total knee arthroplasty: a meta-analysis of randomized controlled trials. J Arthroplasty 2010;25:1240–5. 19837556 10.1016/j.arth.2009.08.013

[38] Webb JM Williams D Ivory JP The use of cold compression dressings after total knee replacement: a randomized controlled trial. Orthopedics 1998;21:59–61. 9474633 10.3928/0147-7447-19980101-14

[39] Volquind D Zardo RA Winkler BC Use of tranexamic acid in primary total knee replacement: effects on perioperative blood loss. Braz J Anesthesiol 2016;66:254–8. 27108821 10.1016/j.bjane.2014.11.004

[40] Wittmann FW Ring PA Blood loss associated with Ring uncemented total knee replacement: comparison between continuous and intermittent suction drainage. J R Soc Med 1984;77:556–8. 6747978 10.1177/014107688407700706 PMC1439930

[41] Woolson ST Perioperative blood loss associated with total knee arthroplasty. A comparison of procedures performed with and without cement. J Bone Joint Surg Am 1991;73:1574. 1748706

[42] Zhang W Liu A Hu DC Effects of the timing of tourniquet release in cemented total knee arthroplasty: a systematic review and meta-analysis of randomized controlled trials. J Orthop Surg Res 2014;9:125. 25467223 10.1186/s13018-014-0125-0 PMC4266218

[43] Olivecrona C Ponzer S Hamberg P Lower tourniquet cuff pressure reduces postoperative wound complications after total knee arthroplasty: a randomized controlled study of 164 patients. J Bone Joint Surg Am 2012;94:2216–21. 23318611 10.2106/JBJS.K.01492

[44] Olivecrona C Blomfeldt R Ponzer S Tourniquet cuff pressure and nerve injury in knee arthroplasty in a bloodless field: a neurophysiological study. Acta Orthop Scand 2013;84:159–64. 10.3109/17453674.2013.782525 PMC3639336 23485070

[45] Jørgensen HR Myoglobin release after tourniquet ischemia. Acta Orthop Scand 1987;58:554–6. 3425287 10.3109/17453678709146398

[46] Rand JA Vascular complications of total knee arthroplasty. Report of three cases. J Arthroplasty 1987;2:89–93. 3612144 10.1016/s0883-5403(87)80014-1

